# How to optimise the coverage rate of infant and adult immunisations in Europe

**DOI:** 10.1186/1741-7015-5-11

**Published:** 2007-05-29

**Authors:** Heinz-J Schmitt, Robert Booy, Robert Aston, Pierre Van Damme, R Fabian Schumacher, Magda Campins, Carlos Rodrigo, Terho Heikkinen, Catherine Weil-Olivier, Adam Finn, Per Olcén, David Fedson, Heikki Peltola

**Affiliations:** 1 Infectious Diseases Service at the Zentrum für Präventive Pädiatrie, Johannes Gutenberg Universität, Langenbeckstr. 1, 55101 Mainz, Germany; 2 National Centre for Immunisation Research & Surveillance (NCIRS), Loked Bag 4001, Westmead NSW 2145, Australia; 3 Stoneyacre, Foxholes Road Horwich BL6 6AL, UK; 4 Centre for the Evaluation of Vaccination, Department of Epidemiology and Social Medicine, University of Antwerp, Universiteitsplein, 1, 2610 Wilrijk, Belgium; 5 Clinica Pediatrica, Universita' degli Studi di Brescia, Piazzale Spedali Civili 1, 25123 Brescia, Italy; 6 Servicio de Medicina Preventiva y Epidemiología, Hospital Vall d'Hebron, Universitat Autònoma de Barcelona, Barcelona 08035, Spain; 7 Hospital Universitari 'Germans Trias I Pujol,' Carretera de Canyet s/n 08916, Barcelona, Spain; 8 Department of Pediatrics, Turku University Hospital, FI-20520 Turku, Finland; 9 Hospital Louis Mourier, Colombres, France; 10 Institute of Child Life and Health, University of Bristol, UK; 11 Department of Clinical Microbiology and Immunology, Örebro University Hospital, Sweden; 12 57 Chemin du Lavoir, 01630 Sergy Haut, France; 13 HUCH Hospital for Children and Adolescents, FI-00029 HUS, Helsinki, Finland

## Abstract

**Background:**

Although vaccination has been proved to be a safe, efficacious, and cost-effective intervention, immunisation rates remain suboptimal in many European countries, resulting in poor control of many vaccine-preventable diseases.

**Discussion:**

The Summit of Independent European Vaccination Experts focused on the perception of vaccines and vaccination by the general public and healthcare professionals and discussed ways to improve vaccine uptake in Europe.

Despite the substantial impact and importance of the media, healthcare professionals were identified as the main advocates for vaccination and the most important source of information about vaccines for the general public. Healthcare professionals should receive more support for their own education on vaccinology, have rapid access to up-to-date information on vaccines, and have easy access to consultation with experts regarding vaccination-related problems. Vaccine information systems should be set up to facilitate promotion of vaccination.

**Summary:**

Every opportunity to administer vaccines should be used, and active reminder systems should be set up. A European vaccine awareness week should be established.

## Background

Compared with other healthcare interventions, vaccination has proved to be one of the most cost-effective health measures of the 20th century, and it is credited with a substantial role in the overall increase in life expectancy. However, despite the proven safety and efficacy of vaccines, immunisation rates remain suboptimal in many European countries, and some common vaccine-preventable diseases are not controlled to the extent to which they could. For example, in 1997, the World Health Organization set the objective of eliminating measles from Europe by 2007. To reach this goal, at least 95% of the population should receive two doses of the measles vaccine. Recent data demonstrate that this is not the case. Measles vaccine coverage remains too low to eliminate the disease, and the virus is still circulating in many western European countries. In 2006, Germany was hit by a large measles epidemic [1], and measles outbreaks, sometimes numbering tens of thousands of cases with substantial numbers of complications and deaths, have also been reported in recent years from other countries such as Switzerland, Spain, the Netherlands, France, and Italy (Schumacher RF, personal communication) [2-7].

Several factors, including inconsistent vaccination systems, lack of political will, and poor understanding or false perceptions of vaccination by the public and healthcare professionals (HCPs) have been identified as barriers to vaccination in Europe [8]. The role of European vaccination policies and systems was discussed by the Summit of Independent European Vaccination Experts (SIEVE) in 2003 [8] (Table 1). In a recent meeting, the group specifically addressed the perception of vaccines and vaccination among the general public and HCPs and discussed how to improve vaccine uptake in Europe. This article summarizes our key findings and gives suggestions for increasing vaccination coverage.

**Table 1:** Society of Independent European Vaccination Experts (SIEVE)

The "Stiftung Präventive Pädiatrie" at the Johannes-Gutenberg-University in Mainz, Germany, has set up a board of independent European vaccination experts who meet at regular "Summits of Independent European Vaccination Experts" to evaluate vaccine needs, current research, and vaccination policies.

To date, SIEVE members have met to discuss vaccination systems in Europe; the epidemiology of measles; the pneumococcal conjugate vaccine; varicella vaccination for all children; and influenza vaccination of children. In addition, the SIEVE steering committee meets at least quarterly to discuss vaccination-related issues and to prepare the group discussions. The articles from each meeting represent the consensus view of the meeting participants.

The current SIEVE steering committee consists of Heikki Peltola (Helsinki; President), Robert Booy (Westmead; Assessor), and Joe Schmitt (Mainz; General Secretary).

## Discussion

### Public perception of vaccines and vaccination

In developed countries, the general public usually has a positive view of vaccines and considers vaccination important. This has been shown in several surveys carried out in Canada [9,10], the USA [11-13], Belgium [14], Germany [15-17], and Italy [18]. In one telephone survey in the USA in 1999, 87% of parents deemed immunisation an extremely important action to keep their children well [11]. A recent survey supported by the European Vaccine Manufacturers (EVM) in five European countries (France, Germany, Italy, the UK, and Spain) revealed a similar attitude: 87% of the 5000 respondents from the general public perceived vaccinations as important, and 82% declared a positive opinion on vaccines [19,20].

A closer look at the results demonstrates, however, that the situation is not as clear-cut as the figures might suggest. A detailed analysis of the EVM survey showed that only 37% of the respondents had a "very positive" perception of the value of vaccinations, whereas 45% of them were more reserved and indicated only a "somewhat positive" perception. A recent study in the US [12] showed that a substantial minority of respondents (15%) considered vaccines unnecessary to prevent certain infectious diseases. According to a Canadian survey [9], 58.7% of respondents agreed that vaccination was among the most cost-effective medical interventions.

Why is the public perception of vaccines so variable? Concerns and misconceptions about vaccines are common and have a negative influence on attitudes towards vaccination. In several studies, fear of side-effects has been expressed as the most frequent reason for not vaccinating children and adults [17,21]. Other reasons include concerns about the safety of the vaccine ingredients, the adequacy of safety testing, and potential severe long-term consequences to children [19-27]. Parents have also expressed concerns about increased adverse events with combination vaccines [19,20,27,28]. Some parents also believe that their children receive too many shots, and that this could weaken the children's immune system, or that the children could cope with disease without immunisation [11,12,24]. These concerns are rarely based on scientific evidence.

A lack of knowledge about vaccines among the general public, especially regarding safety issues, has been confirmed by recent studies. In a Canadian study, one-third of respondents thought that they did not know enough to comment [9]. In a study in Ireland, parents declared that they felt poorly informed about vaccination-related issues [27]. A representative survey carried out in Germany indicated that 50% of parents felt that they were insufficiently informed about vaccination [16].

It is paradoxical that one of the main barriers to vaccination is the huge success of many vaccination programmes. After the near disappearance of the target diseases from everyday life, the diseases and their complications no longer serve as a healthy reminder of the continuing need for prevention through vaccination. Many individuals, HCPs included, have never witnessed the debilitating diseases that the vaccines prevent. This has resulted in increased negligence about vaccination, and the balance has shifted from emphasizing the true benefits of vaccination to increased suspicion of adverse effects of vaccination. Parents who are reluctant to have their children or themselves vaccinated often consider vaccine- preventable diseases as either rare or mild, or irrelevant to their own children [23,25,29,30]. Respondents to the EVM survey were more likely to get vaccinated for travel to a foreign country than for any other reason [19,20]. Infectious diseases were seen as a problem in other regions, but the respondents felt safe within the borders of Europe.

### Role of the media as a source of information

The media plays an important role as a source of information for the general public. However, the quality of the information provided by the media is variable and can sometimes be sensational. Many individuals have strong opinions on vaccinations despite being ill-informed about vaccine-related issues [31]. There is also a small but vociferous anti-vaccination lobby (estimated at 3–5% of the population in Germany [32]) who have quickly adapted to the internet to disseminate their message [33].

The media can have a positive or a negative effect on the public perception of vaccination. Unfortunately, recent vaccination scares have sometimes been mixed by the media with unrelated health scares such as bovine spongiform encephalopathy or blood contaminated with human immunodeficiency virus, which have undermined public faith in government healthcare policies [34]. However, the media could also play a positive role in promoting vaccination and informing the public. An article in the *Sunday Times *that revealed a conflict of interest of the author of a report linking the measles, mumps and rubella (MMR) vaccination to autism did much to reduce the credibility of the author's conclusions in the eyes of the public. However, in many respects, the media were responsible for giving the false alarm about this report in the first place [35].

It must be emphasized that a long-term confidential relationship between medical experts and journalists is of crucial importance. Their relationship is an essential foundation for educating the public in health-related matters.

### Positive communication with the public

Common misconceptions could be overcome by delivering accurate, reliable, and positive information on the benefits of vaccines and the minimal risks associated with their use, as well as increasing awareness of the diseases that the vaccines prevent [36]. Such promotion of information is a vital part of achieving and maintaining high levels of uptake of vaccines.

We identified some key factors in communicating with the general public. First, the public deserves credible and trustworthy information; that is, clear, reliable, and up-to-date data on vaccine-preventable diseases and vaccine safety and efficacy from sources they trust. Second, it is important to communicate positively, not in a "defensive" way or by raising fears about vaccine-preventable diseases. Vaccination should be seen as an initiative for promoting good health, and getting vaccinated could be seen as establishing "peace of mind". For parents, vaccination of children could be viewed as complying with a high standard of childcare and as part of "good parenting practices". Of course, the information should be adapted to the different subsets of the population [37]. Decisions must be made about which groups should be targeted (e.g. parents, adolescents, elderly, or other at-risk groups), and education campaigns must be designed to focus on these specific groups.

Although demographic considerations are important in identifying target groups, individual perspectives and attitudes must also be considered for such an emotive subject as vaccination. For example, four distinct groups of parents have been described on basis of the subjects' attitudes towards vaccination: "vaccine believer", "cautious", "relaxed", and "unconvinced" [38]. Messages customised to these groups could improve the understanding and acceptance of the information on vaccination.

Although education of the general public has been shown to be successful, the most effective methods to be used have not been well elucidated [37]. A combination of various approaches at the European, national, and regional levels is probably necessary, but education will not be fully effective unless there is sufficient enthusiasm and commitment to it at the local level by HCPs and healthcare organisations.

### Role of healthcare professionals

HCPs have a key position in vaccine uptake. They serve as an important source of information for the general public and are the main drivers of vaccination programmes. In the EVM survey [19,20], 68% of respondents cited HCPs as their main information source on vaccines in general, and 81% of parents considered HCPs to be a primary source of information regarding vaccination of their children. In a survey on vaccination in Spain, 69% of respondents cited the paediatrician as their most important source of information [28].

With respect to vaccination, the opinions of HCPs are often more important than the parents' or patients' own points of view. Many parents of fully immunised children express similar attitudes and beliefs to parents of underimmunised children. The determining factor in vaccine acceptance is the HCP's attitude [24]. Physicians' views on vaccine safety are critical in determining whether vaccines will or will not be accepted [39]. The strong link between HCPs' perceptions of vaccination and vaccine uptake has been documented by studies from several countries, including France [19,20], the UK [19,20], Belgium [40], Germany [15,19,20], Italy [18-20], and the USA [11,22,41].

A role in vaccine acceptance is not limited to family physicians and paediatricians. Nurses, pharmacists [42], midwives, and professionals working in various types of childcare centres may also have an important role in educating and informing the general public.

### Information and education of healthcare professionals

HCPs have generally positive attitudes towards vaccination. In the EVM study, 84% of the 800 healthcare professionals interviewed affirmed very positive perceptions of vaccines and the value of vaccination, and another 14% had somewhat positive perceptions [19,20]. However, their level of information on vaccination issues was not always optimum. Surprisingly, education on vaccines and vaccination is poor or nonexistent in the medical curricula in most western European countries. The recent renewal process of the medical curricula at many European universities provides an opportunity to incorporate more extensive training on vaccinology for future generations of physicians. Similar efforts should be made for pharmacy and nursing curricula. A survey to assess the current state of vaccine-related teaching in the formal education of HCPs is recommended as a first step in improving the system.

HCPs often lack precise information on specific issues. In an American study [43], over 90% of 268 physicians interviewed thought that vaccine efficacy was high and that the likelihood of serious side effects was low. However, only 37% could give an accurate estimate of the likelihood that an infant with pertussis would need hospitalisation. Many respondents had inaccurate views of vaccine contraindications. For example, 37% would not administer the MMR vaccine to a child whose mother was pregnant even though administration of a live, attenuated virus vaccine to household members does not present a known hazard to pregnant women [44]. Likewise, 21% would not administer four vaccines simultaneously, despite the fact that such co-administration has been shown to be perfectly safe and effective and is an essential component of childhood vaccination programmes [44].

Most HCPs participating in the EVM study [19,20] were practicing vaccinators, and therefore it is not surprising that they were satisfied with their level of information (satisfaction level of 8.3 on a scale from 1 to 10). Scientific journals were cited as their main source of information. It is remarkable, however, that even among well-informed physicians, there was a need for more information, especially about new vaccines. A study carried out in Ireland revealed that the levels of knowledge about vaccines and vaccine-preventable diseases varied greatly both within and between different groups of HCPs [27]. To varying degrees, HCPs felt that they were ill-equipped to properly inform parents about vaccine-related issues, with those who were less involved in vaccination expressing more concern. They complained about a lack of user-friendly information or the absence of critical information when it was needed most (such as during vaccine scares, when the HCPs are the first-line responders). HCPs all expressed a need for timely and accurate information to help them address parental concerns regarding vaccination [27].

HCPs need rapid access to relevant, up-to-date, and objective information, instead of having to rely on press releases, leaflets from pharmaceutical companies, or the media. One successful initiative in this context is INFOVAC . Created in Switzerland in 2000 and established in France in 2003, INFOVAC is an interactive consultation/information system on vaccines and vaccination for general practitioners and paediatricians. It is run by a network of academic paediatric experts in infectious diseases, who can be contacted at a central e-mail address. HCPs can address every question concerning vaccination and be assured of an answer from an expert within 24–48 hours.

INFOVAC also distributes monthly bulletins on the latest developments in vaccinology [39]. One of the strong points of INFOVAC is that because of its broad academic network it can respond to a wide range of questions from the vaccinators. This successful experience could serve as a model for other countries.

Another successful initiative, started in 2001 by the University of Antwerp, Belgium, is an annual vaccination "Q&A" day. The day is organised without any support from industry in order to avoid any real or perceived bias or conflict of interest. The participating HCPs (on average 350–500 physicians and nurses) set up the programme by sending in their questions in advance, and a panel of local experts is invited to respond. The goal of these annual meetings is to provide up-to-date information for the participants [45].

HCPs not only need information regarding particular vaccines but they also need to be informed about the epidemiology and effect of infectious diseases and about the vaccination rates in their country or region to be able to design appropriate strategies.

### Healthcare professionals need more time to inform the public

In many countries, HCPs act as the principal source of information on vaccines and vaccination for their patients, and thus they are essential for the population at large. Several studies have shown that this places a heavy time burden on the already tight schedules of HCPs. Lack of time is an important cause of missed vaccination opportunities [46]. In one survey, paediatricians, family physicians, and nurses in private practice reported initiating discussion in 70% of visits on the immunisation schedule, common side effects, and when to call the clinic. These HCPs considered lack of time as the greatest barrier to vaccine risk-benefit communication [47]. More time was needed to alleviate parental concerns generated by scare stories [27]. The need to devote attention to treating active medical problems was cited as the most common reason for missed pneumococcal vaccinations in a study of American physicians [48].

Any extra time spent by HCPs addressing vaccine issues is not time wasted. Practices that allowed more time for acute care visits and used more immunisation promotion activities were found to have higher influenza and pneumococcal vaccination rates among adults >65 years of age [46].

### Centralised computer systems and active reminders

Many people do not get fully vaccinated, not because they are against vaccination, but because they tend to forget about it and/or because vaccination schedules are too complex to remember. Active reminder systems such as postcards, telephone calls, or other forms of communication should be set up. Reminders to both patients and providers have been shown to increase vaccination uptake [11,24], but unfortunately these interventions are underused in primary care and even illegal in some countries such as Germany.

Centralised computer systems that would rapidly inform the HCPs about the vaccination status of their patients could substantially promote vaccination. Besides providing important information about the patients to the HCPs, centralised computer systems could be used to provide timely information on local vaccine coverage levels. Availability of reliable data on vaccine coverage is essential for the evaluation of the effectiveness of any intervention strategy [49]. Computers could also be used to help in monitoring the occurrence of vaccine-related adverse events and collecting data on patient response behaviour.

Computerized systems are already in place in some regions of Europe. For example, in the autonomous region of Murcia Province, Spain, 99% of newborn babies are entered into the Registry of the Computerised Vaccination Programme of the Directorate General of Public Health. All parents receive a letter of introduction, a vaccination booklet, and a card with a barcode for each vaccine that the child should receive during the first 2 years. Vaccines administered are recorded on the card, which is connected to the registry managed by the data management centre. This registry provides: (i) a list of properly vaccinated persons; (ii) a list of insufficiently vaccinated persons who are periodically reminded by mail or telephone of the convenience of keeping up with the schedule; and (iii) a certificate of vaccination status [28].

In Flanders, Belgium, a pilot electronic vaccination database, Vaccinnet, was set up in 2003 for infants visiting "well baby" clinics [50]. By 2006-7, this database will cover all infants, and it will later expand to cover all childhood and adolescent vaccines administered by general practitioners, paediatricians, and school physicians, plus adult vaccines given by general practitioners and occupational health physicians. Vaccinnet aims to avoid the overuse of vaccines, identify pockets of vaccine underuse, record vaccine coverage, and evaluate the success of vaccination programmes. Vaccinnet is a central ordering system for vaccines and can be used as a  system to the record coverage and to report adverse events.

### Increased opportunities for vaccination

Every opportunity to administer vaccines should be used. For example, patients discharged from hospital for pulmonary disease should be offered immediate injection of pneumococcal vaccine and, in the autumn, of influenza vaccine. MMR vaccine should be offered to seronegative women when they are discharged from the maternity ward. Easy access to vaccination in non-traditional settings should also be considered. Depending on national and local circumstances, vaccinations could be performed in settings such as childcare centres and nursing homes and during home visits. In addition, where available, access to vaccination centres should be facilitated, with evening and weekend opening hours. Increasing opportunities for vaccination will increase vaccine uptake.

Standing orders that authorise nurses and pharmacists to administer vaccines without a physician contact according to an institution-approved or physician-approved protocol have proved successful in increasing vaccination rates in adults [51]. They were found to be more effective than computerised reminders for increasing both influenza and pneumococcal vaccine administration [52]. They can be implemented in inpatient and outpatient facilities and other non-traditional environments, greatly increasing the opportunities for vaccination.

## Summary

### Conclusion

Vaccination rates in Europe are not what they should or could be. Public perception of vaccination is important, but rather than concentrating on failures of vaccination it might be better to identify and implement strategies that focus on HCPs and that have been shown to work.

Clearly, HCPs play a central role in vaccine uptake. They remain the most important sources of information to the public and the most important advocates of vaccination although their contributions are often unappreciated and underused. To feel confident in carrying out their responsibilities, they need the support of health authorities and they also need to be sufficiently informed about vaccine-related issues and problems. The INFOVAC network is a good step in this direction, and it could be expanded to other countries. In addition, medical education should be revised to include additional training in vaccinology in the medical, pharmaceutical, and nursing curricula. The general public could also benefit from better education. Many of the fears and concerns of lay persons could be overcome by easy access to accurate information presented in an understandable format.

Even if all the information would be available, however, the lack of time for communication remains a great barrier to vaccination and must be addressed. Moreover, once the idea of vaccination is accepted, the opportunities for administering the injections themselves need to be increased.

Many of the issues highlighted here could be addressed during a European Vaccination Awareness Week. Such an event could involve the media to inform the general public, to raise awareness, and to promote dialogue between HCPs and the public. Such events have been successfully implemented in the USA ("National Immunization Awareness Month [53]) and Canada (National Immunization Awareness Week [54]. The Pan American Health Organisation has organised an international event (Vaccination Week in the Americas [55]). Such initiatives focus the attention of the public on the importance of vaccinations. National and Europe-wide organisations could promote and support community-based actions to inform people at the local level. These would encourage people to check their vaccination status, educate themselves on vaccination issues, and perhaps even get vaccinated.

## Competing interests

SIEVE is supported by an unrestricted educational grant from the Stiftung Präventive Pädiatrie at the Johannes-Gutenberg-University, Mainz, Germany. There was no other funding source.

## Authors' contributions

All authors contributed to the manuscript by a country-specific presentation and by discussing the topic at a 2-day meeting held in Frankfurt, November 12–14 (2004). Following the meeting, the manuscript was prepared by a science writer (Dr Britta Gröndahl) employed by the University of Mainz at the department of the first author. The first author was in charge of producing the final version of the manuscript with input via "electronic discussions".

**Table 2:** Suggestions from SIEVE

• Implement an integrated advocacy and communication programme to inform the public.
• Promote the training of HCPs not only in the scientific and medical aspects of vaccination but also in the management of vaccination programmes.
• Conduct a survey of vaccinology education in the curricula of HCPs in European countries and make recommendations based on the results.
• Extend and support an INFOVAC-like network in Europe.
• Provide support to HCPs for increasing immunisation rates in their patients:
◦ By allocating them sufficient time during their patients' consultation to inform them about vaccination.
◦ By setting up computerized information systems for HCPs and providing them with training that will allow them to record their patients' vaccination status and implement active reminder programmes.
•Increase vaccination opportunities (e.g., standing orders, more convenient opening hours).
• Support the organisation of a "European Immunisation Awareness Week". HCP, healthcare professional.

## Pre-publication history

The pre-publication history for this paper can be accessed here:


